# Laser-Induced Liquid-Phase Boron Doping of 4H-SiC

**DOI:** 10.3390/ma18122758

**Published:** 2025-06-12

**Authors:** Gunjan Kulkarni, Yahya Bougdid, Chandraika (John) Sugrim, Ranganathan Kumar, Aravinda Kar

**Affiliations:** 1 Electrical and Computer Engineering Department, University of Central Florida, Orlando, FL 32816, USA; gunjan.kulkarni@ucf.edu (G.K.); chandraika.sugrim.civ@us.navy.mil (C.S.); 2 Center for Research and Education in Optics and Lasers, University of Central Florida, Orlando, FL 32816, USA; yahya.bougdid@ucf.edu; 3 Mechanical and Aerospace Engineering Department, University of Central Florida, Orlando, FL 32816, USA; ranganathan.kumar@ucf.edu; 4 Naval Air Warfare Center—Aircraft Division (NAWCAD), Patuxent River, MD 20670, USA

**Keywords:** laser doping, silicon carbide, boron, p–n junction, refraction index

## Abstract

4H-silicon carbide (4H-SiC) is a cornerstone for next-generation optoelectronic and power devices owing to its unparalleled thermal, electrical, and optical properties. However, its chemical inertness and low dopant diffusivity for most dopants have historically impeded effective doping. This study unveils a transformative laser-assisted boron doping technique for n-type 4H-SiC, employing a pulsed Nd:YAG laser (λ = 1064 nm) with a liquid-phase boron precursor. By leveraging a heat-transfer model to optimize laser process parameters, we achieved dopant incorporation while preserving the crystalline integrity of the substrate. A novel optical characterization framework was developed to probe laser-induced alterations in the optical constants—refraction index (n) and attenuation index (k)—across the MIDIR spectrum (λ = 3–5 µm). The optical properties pre- and post-laser doping were measured using Fourier-transform infrared spectrometry, and the corresponding complex refraction indices were extracted by solving a coupled system of nonlinear equations derived from single- and multi-layer absorption models. These models accounted for the angular dependence in the incident beam, enabling a more accurate determination of n and k values than conventional normal-incidence methods. Our findings indicate the formation of a boron-acceptor energy level at 0.29 eV above the 4H-SiC valence band, which corresponds to λ = 4.3 µm. This impurity level modulated the optical response of 4H-SiC, revealing a reduction in the refraction index from 2.857 (as-received) to 2.485 (doped) at λ = 4.3 µm. Structural characterization using Raman spectroscopy confirmed the retention of crystalline integrity post-doping, while secondary ion mass spectrometry exhibited a peak boron concentration of 1.29 × 10^19^ cm^−3^ and a junction depth of 450 nm. The laser-fabricated p–n junction diode demonstrated a reverse-breakdown voltage of 1668 V. These results validate the efficacy of laser doping in enabling MIDIR tunability through optical modulation and functional device fabrication in 4H-SiC. The absorption models and doping methodology together offer a comprehensive platform for paving the way for transformative advances in optoelectronics and infrared materials engineering.

## 1. Introduction

Silicon carbide (SiC), particularly the 4H polytype, has emerged as a cornerstone material in wide-bandgap semiconductor research due to its exceptional electrical, thermal, and mechanical properties, including high breakdown electric field, wide bandgap (3.23 eV), excellent thermal conductivity, and chemical stability [1,2,3,4]. These attributes make 4H-SiC indispensable for power electronics, high-frequency communication systems, and optoelectronic devices operating in extreme environments [5,6,7,8]. Additionally, its potential extends to advanced applications such as spin qubits in quantum computing [9], high-voltage insulation [10], and biosensing technologies [11], and its application in high-temperature and radiation-resistant environments such as aerospaces and other extreme conditions [12], reinforcing the need for precise control over its electrical and optical characteristics through advanced material processing techniques. Its exceptional combination of electrical, thermal, and mechanical properties makes SiC indispensable for high-performance applications, including gas sensors [1], power metal–oxide semiconductor field-effect transistors (MOSFETs) [3], light-emitting diodes (LEDs) [13], and p-n junction diodes [2,14].

Despite these advantages, SiC doping remains a significant challenge due to its extreme hardness and strong chemical inertness, as well as the inherently low diffusivity of most dopant species [3]. Conventional doping techniques—such as ion implantation, thermal diffusion, and epilayer doping—are often constrained by critical drawbacks, including the requirement for elevated processing temperatures, the introduction of implantation-induced lattice damage, and limitations in dopant solubility within the SiC matrix [3]. In contrast, laser doping has emerged as a compelling alternative, offering localized, non-equilibrium energy delivery that facilitates effective dopant incorporation while mitigating substrate damage. This approach supports selective-area doping, rapid thermal cycling, and the ability to surpass equilibrium solubility limits, thereby enhancing its potential for advanced semiconductor applications [15,16,17]. By employing high-intensity laser irradiation, dopants are introduced directly into the substrate, enabling the precise modulation of the material’s electrical and optical characteristics through controlled thermal and chemical dynamics. Compared with conventional techniques, laser doping provides significant advantages, including improved dopant distribution uniformity, and fine-tuned depth control. Moreover, it enables cost-efficiencies by eliminating the necessity for prolonged high-temperature or vacuum-based treatments [13]. These attributes make laser doping particularly advantageous for the fabrication of next-generation power electronic devices, photonic components, and sensors, where precise doping control in wide-bandgap semiconductors such as SiC is critical for achieving optimal performance and reliability.

As a thermodynamically non-equilibrium process, the laser doping of SiC involves supplying high energy to a specific region of the substrate, inducing transient heating while maintaining the rest of the substrate at an ambient temperature [15]. This localized energy delivery significantly enhances dopant diffusion into the SiC lattice, positioning laser doping as an effective technique for precise, spatially controlled dopant incorporation. The technique has been successfully employed to introduce various dopant species into SiC, including gallium (Ga) [1], phosphorus (P) [2,14], nitrogen (N) [3], aluminum (Al) [3,16], chromium (Cr) [13], and boron (B) [13], enabling the development of high-performance SiC-based microelectronic devices. In contrast to conventional doping techniques that rely on solid, liquid, or gaseous precursors and often involve complex micro- or nano-fabrication steps, laser doping using solution precursors presents several distinct advantages. Solution-based precursors can provide a higher dopant concentration at the substrate surface, while offering superior safety and environmental compatibility due to their non-pyrophoric, non-toxic, and ambient-stable nature [14,15,16]. As a result, liquid-phase laser doping—where the SiC substrate is immersed in a dopant-containing solution and subsequently irradiated—emerges as a highly promising strategy for the selective doping of both p-type and n-type SiC substrates [14,16]. Moreover, laser doping allows for reproducible and tunable control over dopant concentration and depth profiles through adjusting laser process parameters and substrate characteristics [16]. Compared to traditional approaches, such as thermal diffusion, epilayer doping, and ion implantation, laser doping with solution precursors offers a streamlined and efficient alternative. It eliminates the need for high-temperature annealing and complex multi-step fabrication, thereby reducing the processing time, simplifying the workflow, and lowering the manufacturing costs. Collectively, this method enhances process flexibility, environmental sustainability, and precision in doping control, positioning it as a superior technique for advanced SiC device fabrication.

During the laser doping process, two key physical phenomena occur simultaneously: the laser-induced heating of the SiC substrate and the diffusion of dopant atoms into the substrate [1,2]. The laser significantly increases the temperature at the laser–substrate interaction zone, though it remains below the SiC peritectic temperature (2830 °C) [13], leading to localized melting and the subsequent re-solidification of the SiC substrate surface [1,2,3]. This rapid heating generates thermal gradients that promote the diffusion of dopant atoms into the substrate. As the laser continues to irradiate the surface, the dopant atoms migrate into the substrate through temperature-driven diffusion. The interaction of these processes results in the incorporation of dopant atoms into the substrate’s crystal structure, which in turn alters its electrical and optical properties. Lim et al. [1] developed an uncooled MIDIR detector through utilizing the laser doping of n-type 4H-SiC with triethyl-gallium [Ga(C_2_H_5_)_3_] as a Ga precursor. Their investigation unveiled the diffusion of Ga atoms into n-type 4H-SiC, creating an acceptor energy level of 0.3 eV within the SiC bandgap, corresponding to an MIDIR wavelength of λ = 4.21 µm. An analysis using FTIR spectrometry in [1] confirmed the integration of Ga atoms into the n-type 4H-SiC substrate. The exposure of Ga-doped 4H-SiC to λ = 4.21 µm caused alterations in electron density, thus affecting its transmittance, reflectance, absorptance, and refraction index. It was reported in [1] that the refraction index of SiC decreases after laser doping, reducing from 3.442 to 3.217. After doping, some dopants may occupy interstitial sites within the SiC lattice, altering the electronic structure and impacting the acceptor energy levels [1]. When dopant atoms occupy substitutional sites, they can replace silicon or carbon atoms, creating a p-type semiconductor with multiple acceptor levels. Furthermore, differences in atomic size between the dopants and the lattice atoms can introduce mechanical stress. Based on a thermal diffusion model, Bet et al. [13] calculated the diffusivity of Cr atoms at different depths within the doped SiC substrates as a function of temperature induced by laser. They reported a maximum diffusivity of Cr atoms in SiC of 6.9 × 10^−12^ cm^2^/s at 3046 K for 6H-SiC and 4.61 × 10^−10^ cm^2^/s at 2898 K for 4H-SiC substrates. Thermal doping techniques are inherently limited by the solid solubility limits of the material, while ion implantation can surpass these limits but often causes significant damage to the implanted wafer [13]. In contrast, laser doping, a non-equilibrium process, enables the solubility limits to be exceeded with minimal damage. This extended solubility has been observed for dopants such as Al, N and B, as addressed in [1,3,13]. Several factors contribute to this increased solubility: (i) surface interactions reduce the stress caused by atomic size mismatches between C, Si, and dopant atoms; (ii) atomic reconstruction near the surface generates stress fields; and (iii) the pulsed laser’s rapid heating and cooling cycles trap dopants by causing the lattice to expand and contract quickly. The combined effects of impurity-induced stress and spatial variations in surface and thermal stress significantly lower the energy required for dopants to occupy specific lattice sites in the SiC substrate. Barkby et al. [17] introduced a single-step method called high-pressure gas-immersion excimer laser doping (HP-GIELD) for creating nitrogen-hyperdoped silicon. This technique offers ultrafast processing, scalability, precise control, and excellent reproducibility. Using HP-GIELD, they successfully produced nitrogen-hyperdoped silicon in nitrogen-rich environments. By optimizing the process parameters, they achieved nitrogen concentrations exceeding 3.01 × 10^21^ cm^−3^, which is 12 times higher than the previously reported maximum dopant concentration and six orders of magnitude higher than the solid solubility limit [17]. Laser doping enabled the formation of interconnects on insulating SiC surfaces without requiring a metallization process [18,19]. Consequently, it presents a potential alternative to conventional doping methods [3], especially for wide-bandgap semiconductor materials.

Excimer lasers, such as ArF (λ = 193 nm), KrF (λ = 248 nm), and XeCl (λ = 308 nm), have been widely explored for doping SiC substrates. However, these lasers induce strong photon–material interactions, leading to excessive localized heating and surface ablation. This can compromise the structural integrity of SiC and degrade its electrical properties, making excimer lasers less applicable for doping [2,14,16]. In contrast, the Nd:YAG laser (λ = 1064 nm) offers several advantages over excimer lasers for doping. Its lower photon energy and deeper dopant penetration enables higher surface temperatures and deeper heat conduction into the substrate, which is beneficial for efficient dopant incorporation [3]. Additionally, the longer irradiation time associated with the Nd:YAG laser helps maintain an elevated substrate temperature, thereby enhancing the diffusion of boron atoms into SiC. Previous studies have employed the Nd:YAG laser for doping with conventional precursors [1,13], but these approaches have struggled to achieve high and deep dopant incorporation. To address these limitations, this work investigates the use of an NIR pulsed Nd:YAG laser (λ = 1064 nm) in combination with a solution precursor. This approach has the potential to significantly enhance dopant penetration and improve process efficiency, offering a more effective method for the laser doping of SiC. Aqueous boric acid (H_3_BO_3_) solution was chosen as the boron precursor in the current study. A heat-transfer model was used to guide the selection of appropriate laser process parameters for doping crystalline 4H-SiC substrates, ensuring that phase transformations are avoided. FTIR spectrometry was conducted within the MIDIR range to analyze the changes in the optical properties, specifically the reflectance and absorptance of the as-received and B-doped 4H-SiC substrates. The absorption peak at λ = 4.3 µm, corresponding to the acceptor level at 0.29 eV created by boron atoms within the 4H-SiC bandgap, was identified. The absorption models developed in the current study were presented to determine the refraction and attenuation indices before and after doping. The results obtained from the absorption models were compared with those obtained using Fresnel’s method [15]. Additionally, the impact of variations in the incident angle on the measured reflectance and their effects on the optical properties are reported. The absorption models outlined in this paper are essential for precisely assessing the optical properties of SiC in laser doping technologies.

In prior research [15], we demonstrated the feasibility of laser doping 4H-SiC using a solution-based precursor and analyzed its influence on the SiC’s optical properties using Fourier-transform infrared (FTIR) spectrometry at normal incidence. However, this earlier study was limited in scope, focusing solely on optical responses and employing Fresnel’s equation without independent validation for determination of the refraction index. Furthermore, no electrical characterization or comparative assessment against conventional doping techniques was undertaken. The present study significantly advances beyond our previous work by integrating both optical and electrical characterizations of laser-doped 4H-SiC, thereby offering a more holistic evaluation of the doping process. Specifically, we introduced robust, angle-resolved absorption models to accurately extract the complex refraction index (n + ik) of as-received and boron-doped SiC, validated against Fresnel’s method. These models allow for the precise determination of optical constants under varying incidence angles, providing a more comprehensive understanding of doping-induced modifications. Crucially, this work includes a detailed electrical characterization of a laser-fabricated p–n junction diode, demonstrating the functional viability of the laser doping approach. The diode exhibited a reverse-breakdown voltage of 1668 V, and temperature-dependent resistivity measurements confirmed the electrical activation of the boron dopant. Secondary ion mass spectrometry (SIMS) analysis revealed a peak boron concentration of 1.29 × 10^19^ cm^−3^ and a junction depth of 450 nm, indicating effective dopant diffusion. This study not only refines the methodology for optical property extraction in laser-doped SiC but also validates the efficacy of the doping process through the electrical performance metrics of fabricated devices. By addressing both optical and electrical domains, and comparing the developed technique with established doping methods, this work provides a comprehensive platform for advancing SiC-based optoelectronic and power device technologies.

## 2. Process Parameters Selection

To enable the effective laser doping of 4H-SiC, it is critical to optimize laser process parameters to enhance dopant diffusion while preventing thermal damage or phase transitions. This requires precise control of the laser power, irradiation duration, and optical absorption to ensure that the surface temperature stays below the peritectic point of SiC (2830 °C) [13]. To support this analysis, the optical properties of as-received n-type 4H-SiC substrate at λ = 1064 nm were characterized using a Lambda 365+ UV–Vis spectrophotometer (Perkin Elmer, Inc., Waltham, MA, USA). The measured transmittance (T = 18.05%), reflectance (R = 35.13%), and absorptance (A = 46.79%) at λ = 1064 nm provide critical input for estimating the laser process parameters corresponding to the optimal theoretical doping temperature. In particular, the absorptance of the SiC substrate directly governs the energy coupling at the laser–substrate interaction zone, thereby strongly influencing the peak temperature reached during irradiation [15].

**Figure 1:**
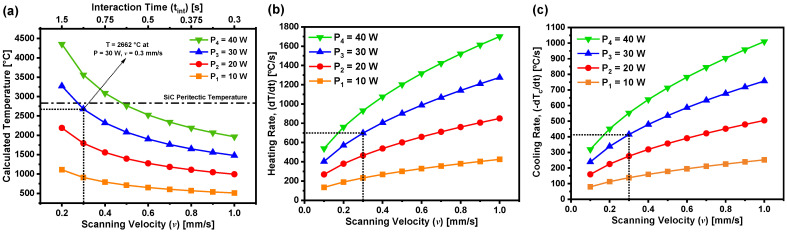
(**a**) Temperature at the laser–SiC substrate interaction zone, (**b**) heating rate, and (**c**) cooling rate as a function of scanning velocity for various laser powers.

The temperature at the laser–substrate interaction zone can be estimated by incorporating parameters such as laser power, spot size, the absorptance of 4H-SiC at λ = 1064 nm, the thermal conductivity, the thermal diffusivity, and the laser–substrate interaction time [15]. A comprehensive heat transfer model was employed to compute the doping temperature as a function of laser power and substrate scanning velocity, using the thermophysical and optical properties of the SiC substrate [20]. The calculated surface temperature, shown in Figure 1a, varies with both laser power and scanning speed. The results demonstrate that lower scanning velocities facilitate increased thermal exposure, thereby reducing thermal stress while enabling efficient dopant diffusion without inducing structural damage or phase transitions. To ensure substrate integrity during laser doping, it is also critical to evaluate the heating rate, defined as the temporal rate of temperature rise at the laser–substrate interface, $\left(\frac{dT(0,t)}{dt}\right)$. The heating rate of the substrate at any time t = t_int_, where t_int_ is the interaction time, is determined by Equation (1). In Equation (1), T represents the temperature, A is the absorptance of SiC at λ = 1064 nm, P is the irradiation laser power, ᴋ is the thermal conductivity, a is the area of laser–SiC substrate, κ is the thermal diffusivity, v is the scanning velocity, and d is the diameter of the laser spot [20,21]:

(1) $$\frac{dT}{dt}=\left(\frac{AP}{ᴋa}\right)\sqrt{\frac{\kappa v}{\pi d}}$$

The overall scenario, which encompasses both spatial and temporal changes in the substrate temperature during the doping process, can be described using Equation (2), obtained from [20,21]:

(2) $$T\left(z,t\right)=\left\{\frac{2Pe^{-\left[\frac{z^{2}}{4\kappa t}\right]}}{ᴋa}\sqrt{\frac{\kappa t}{\pi }}\right\}-\left\{\frac{Pz}{a}erfc\left[\frac{z}{2\sqrt{\kappa t}}\right]\right\}$$

Here, the vertical axis, denoted as z, is oriented downwards and originates from the center of the laser beam. By considering $T\left(z,t_{int}\right)$ in Equation (2) as the initial condition, the solution to the one-dimensional transient heat conduction equation (Equation (1)) depicts the temperature distribution, $T_{c}\left(z,t\right)$, within the material during the cooling phase after the laser beam has heated the interaction zone, at which point doping can be induced. To obtain Equation (3), the derivative of $T_{c}\left(z,t\right)$ with respect to time is evaluated at z = 0 and t = t_int_; this signifies the cooling rate at the specific t_int_ [20,21].

(3) $$-\frac{{dT}_{c}}{dt}=\left(\frac{0.293P}{ᴋa}\right)\sqrt{\frac{\kappa v}{\pi d}}$$

The heat-transfer model was previously validated using a simplified approach [21] to predict laser-induced temperature profiles during the sintering of TiO_2_ nanoparticles (NPs) on quartz substrates. The model accurately forecasted the anatase-to-rutile phase transformation, predicting a temperature range of 550–600 °C under laser irradiation at 3.55 W and a scanning speed of 2 mm/s. These conditions corresponded to the experimentally observed phase change, as evidenced by the formation of rutile-phase TiO_2_ and the characteristic brown coloration shown in Figure 8g of [21]. This validation established the model’s effectiveness in reliably correlating laser process parameters with phase transitions.

Building on the validated framework, this study employs the heat-transfer model to optimize boron doping in 4H-SiC by predicting the substrate’s thermal response under various laser powers and scanning velocities, minimizing experimental iterations and preventing thermal degradation. Laser-induced heating facilitates boron incorporation through localized thermal activation, focused around a 300 µm laser spot. Crucially, the model ensures that the doping temperature remains below the SiC peritectic temperature (2830 °C), thereby preventing crystalline phase transformations. Among the tested conditions, laser powers of 10 W and 20 W yielded estimated surface temperatures of 910 °C and 1794 °C, respectively, which were insufficient for effective boron diffusion. A laser power of 30 W was found to be optimal, producing a localized temperature of ~2662 °C—this is adequate to allow for the substitutional diffusion of boron atoms into the SiC lattice (Figure 1b). Additionally, the model predicted that lower scanning velocities reduce cooling rates, minimizing thermal gradients that could induce mechanical stress. For instance, at 40 W and a scanning speed of 1 mm/s, the model indicated a rapid thermal rise followed by a steep cooling, increasing the risk of substrate cracking (Figure 1c). As a result, a power–speed combination of 30 W and 0.3 mm/s was selected to ensure sufficient thermal energy for doping, while enabling controlled cooling to prevent substrate breakage. By establishing a quantitative link between laser parameters and thermal behavior, the model facilitates precision control over dopant incorporation and structural integrity. This predictive capability enhances process reliability and reduces the need for extensive empirical tuning, underscoring the model’s value in advanced material processing.

## 3. Experimental Procedure

### 3.1. SiC Substrate Cleaning Procedure and Preparation of Dopant Solution

An n-type 4H-SiC wafer with a thickness of D = 350 μm and nitrogen dopant concentration (N_D_) of ~10^16^ cm^−3^ was procured from II-VI Inc. (Pine Brook, NJ, USA) and diced to produce 10 × 10 mm^2^ substrates. Initially, the SiC substrates were scrubbed with soap and rinsed thoroughly with deionized (DI) water. They were then immersed in a 1:1 mixture of 98% sulfuric acid (H_2_SO_4_) and 30 wt.% hydrogen peroxide (H_2_O_2_) for 15 min, followed by another rinse with DI water. Next, the substrates were treated with a 6:1 buffered oxide etchant (BOE) for 10 min to eliminate any native silicon dioxide (SiO_2_) on their surfaces. After rinsing with DI water, they were cleaned with acetone and methanol, rinsed again with DI water, and finally dried with nitrogen (N_2_) gas.

The dopant solution was formulated using boric acid (H_3_BO_3_) powder as the precursor. All chemicals used in this study were purchased from Sigma-Aldrich, Inc. (Burlington, MA, USA). An aqueous boric acid solution with a concentration of 4.63 wt.% (equivalent to 0.75 M) was prepared, yielding a boron concentration of 4.52 × 10^20^ cm^−3^. This formulation deliberately surpasses the established solid solubility limit of boron in SiC (2.5 × 10^20^ cm^−3^) [13], ensuring a high density of boron dopants is supplied to the substrate surface before laser-assisted doping.

### 3.2. Optical Setup for Laser Doping

**Figure 2:**
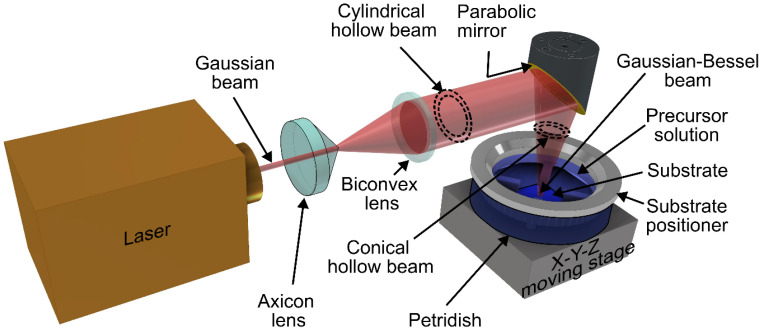
Diagrammatic representation of the optical configuration employed for laser doping of the 4H-SiC substrate.

The schematic of the optical setup for laser doping is shown in Figure 2. The Nd:YAG laser (Lee Laser Inc., Model: LEE-8150MQ, Orlando, FL, USA) employed, with a 170 ns pulse width and a 30 kHz repetition rate, enabled prolonged irradiation and maintained an elevated substrate temperature, thereby facilitating the diffusion of boron atoms into SiC. The Gaussian beam from the laser system was transformed into a cylindrical annular beam using an arrangement of axicon and biconvex lenses. It was then focused into a hollow laser cone using a gold-coated parabolic mirror, resulting in a Gaussian–Bessel beam of ~300 µm in diameter. The interaction between the laser beam, dopant solution, and substrate occurred at the apex of the cone. The laser beam was scanned across a 6 × 6 mm^2^ area of the SiC substrate using a motorized 3D stage. Laser irradiation can induce the formation of additional vacancies in SiC as a result of stress mechanisms such as thermal expansion, shock wave generation, and vibrational excitation. Thus, the 4H-SiC substrate was irradiated at ambient temperature with 30 W power and 0.3 mm/s scanning velocity to create vacancies by out-diffusing nitrogen atoms from the SiC surface and to promote boron diffusion. Doping was carried out on the Si-face of the substrate with a 1 sec interaction time per scan. Subsequently, the substrate was immersed in the dopant solution, where the thickness of the solution layer above the SiC surface was carefully adjusted to ~0.1 mm to avoid interference with the laser doping process due to absorption [22].

During the laser doping process, the 4H-SiC substrate was submerged in the dopant solution and subjected to laser irradiation. The localized heating induced by the laser resulted in the thermal decomposition of the dopant solution at the laser-heated spot, generating boron atoms that subsequently diffused into SiC. According to the heat-transfer model described in Section 2, the doping temperature briefly reached 2662 °C at the applied power of 30 W and 0.3 mm/s speed, remaining below the SiC peritectic threshold (2830 °C). The rapid heating facilitated boron diffusion while the laser pulse was ON, and when the laser pulse was OFF, the substrate underwent rapid cooling, effectively immobilizing the boron dopant atoms within the SiC lattice. Post-doping, the substrate was cleaned with an aqueous 45 wt.% potassium hydroxide (KOH) solution, followed by rinsing with acetone, methanol, and water, and was then dried with N_2_ gas, respectively.

## 4. Absorption Models for the Determination of Refraction and Attenuation Indices Pre- and Post-Laser Doping

Hecht [23] presented the electromagnetic theory of light reflection and transmission through both single and multilayer films composed of homogeneous, non-magnetic, and linear isotropic media. The transfer matrix method in [23] for calculating the reflection and transmission coefficients incorporates both the real and imaginary components of the refraction index. This method proves particularly effective for analyzing variations in reflection coefficients due to differing refraction indices across multiple layers. Building on Hecht’s foundational work, we developed absorption models to calculate the real (refraction index, n) and imaginary (attenuation index, k) components of the complex refraction index of SiC substrates, both before and after laser doping. In this section, we present the single- and multi-layer absorption models used to determine n and k for the as-received (undoped) and doped regions of the SiC substrates. These models utilize FTIR spectrometry measurements at various incident angles to evaluate the optical properties of both the undoped and doped regions of the SiC substrate.

**Figure 3:**
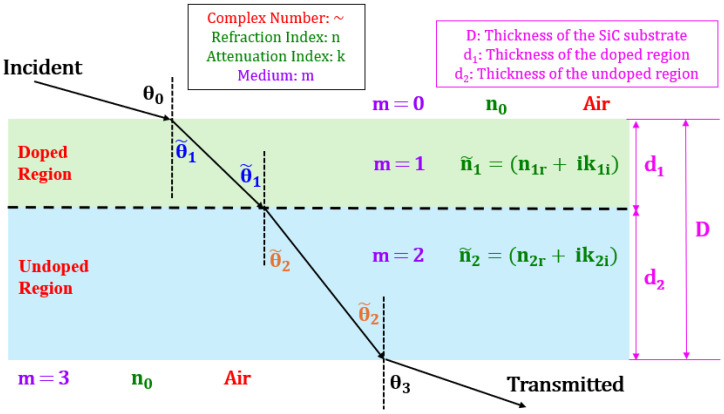
Schematic of light propagation through doped and undoped regions of the SiC substrate.

In Figure 3, $n_{0}$ denotes the refraction index of air (media, m = 0 and m = 3), $n_{1}$ and $k_{1}$ denote the refraction and attenuation indices of the doped region (medium, m = 1) of the substrate, and $n_{2}$ and $k_{2}$ represent the refraction and attenuation indices for the undoped region (medium, m = 2). The angles $\theta_{0}$ and $\theta_{3}$ are the known real angles from experimental conditions, while ${\tilde{\theta }}_{1}$ and ${\tilde{\theta }}_{2}$ are complex angles for oblique incidence. Complex angles represent the effective angles of refraction or transmission in lossy materials, combining phase rotation (real part) and attenuation due to absorption (imaginary part) [24]. The real part indicates how the wave’s direction changes, while the imaginary part describes the reduction in amplitude from absorption. This concept is crucial for understanding wave behavior in materials such as doped 4H-SiC substrates, where both refracted angle and attenuation must be considered.

### 4.1. Single-Layer Absorption Model for Determining $n_{2}$ and $k_{2}$ for the Undoped SiC Region

The optical properties of the undoped SiC region were determined using the absorption model outlined below. The relationship between the reflection coefficient (r), wavelength (λ) and angle of incidence (θ) for both perpendicular (Equation (4)) and parallel polarizations (Equation (5)) can be expressed as follows [25]:

(4) $$r_{s}\;=\frac{a^{2}+b^{2}-2a\;cos\theta +\cos^{2}\theta }{a^{2}+b^{2}+2a\;cos\theta +\cos^{2}\theta }$$

(5) $$r_{p}=\frac{a^{2}+b^{2}-2a\;sin\theta \;tan\theta +{sin}^{2}\theta \;{tan}^{2}\theta }{a^{2}+b^{2}+2a\;sin\theta \;tan\theta +{sin}^{2}\theta \;{tan}^{2}\theta }\;{[r}_{s}]$$

where

(6) $$a=\frac{1}{\sqrt{2}}{\left\{{\left[{\left(n_{2}^{2}-k_{2}^{2}-{sin}^{2}\theta \right)}^{2}+4\;n_{2}^{2}k_{2}^{2}\right]}^{\frac{1}{2}}+n_{2}^{2}-k_{2}^{2}-{sin}^{2}\theta \right\}}^{\frac{1}{2}}$$

(7) $$b=\frac{1}{\sqrt{2}}{\left\{{\left[{\left(n_{2}^{2}-k_{2}^{2}-{sin}^{2}\theta \right)}^{2}+4\;n_{2}^{2}k_{2}^{2}\right]}^{\frac{1}{2}}-n_{2}^{2}+k_{2}^{2}+{sin}^{2}\theta \right\}}^{\frac{1}{2}}$$

The reflectance of the undoped SiC region $\left(R_{U}\right)$ is determined by averaging the perpendicular and parallel polarization components of r, and is expressed as follows:

(8) $$R_{U}=\frac{r_{s}+r_{p}}{2}$$

The simplified expression for $R_{U}$ is obtained by expressing it in terms of $n_{2}$ and $k_{2}$, as follows:

(9) $$\begin{array}{c} 2R_{U}=\left[\frac{{\left[{\left(n_{2}^{2}-k_{2}^{2}-{sin}^{2}\theta \right)}^{2}+4n_{2}^{2}\;k_{2}^{2}\right]}^{½}-\left(\sqrt{2}{\left[{\left[{\left(n_{2}^{2}-k_{2}^{2}-{sin}^{2}\theta \right)}^{2}+4n_{2}^{2}\;k_{2}^{2}\right]}^{½}+n_{2}^{2}-k_{2}^{2}-{sin}^{2}\theta \right]}^{\frac{1}{2}}\times cos\theta \right)+{cos}^{2}\theta }{{\left[{\left(n_{2}^{2}-k_{2}^{2}-{sin}^{2}\theta \right)}^{2}+4n_{2}^{2}\;k_{2}^{2}\right]}^{½}+\left(\sqrt{2}{\left[{\left[{\left(n_{2}^{2}-k_{2}^{2}-{sin}^{2}\theta \right)}^{2}+4n_{2}^{2}\;k_{2}^{2}\right]}^{½}+n_{2}^{2}-k_{2}^{2}-{sin}^{2}\theta \right]}^{\frac{1}{2}}\times cos\theta \right)+{cos}^{2}\theta }\right]\times \\ \left\{\frac{{\left[{\left(n_{2}^{2}-k_{2}^{2}-{sin}^{2}\theta \right)}^{2}+4n_{2}^{2}\;k_{2}^{2}\right]}^{½}-\left(\sqrt{2}{\left[{\left[{\left(n_{2}^{2}-k_{2}^{2}-{sin}^{2}\theta \right)}^{2}+4n_{2}^{2}\;k_{2}^{2}\right]}^{½}+n_{2}^{2}-k_{2}^{2}-{sin}^{2}\theta \right]}^{\frac{1}{2}}\times sin\theta \;tan\theta \right)+{sin}^{2}\theta \;{tan}^{2}\theta }{{\left[{\left(n_{2}^{2}-k_{2}^{2}-{sin}^{2}\theta \right)}^{2}+4n_{2}^{2}\;k_{2}^{2}\right]}^{½}+\left(\sqrt{2}{\left[{\left[{\left(n_{2}^{2}-k_{2}^{2}-{sin}^{2}\theta \right)}^{2}+4n_{2}^{2}\;k_{2}^{2}\right]}^{½}+n_{2}^{2}-k_{2}^{2}-{sin}^{2}\theta \right]}^{\frac{1}{2}}\times sin\theta \;tan\theta \right)+{sin}^{2}\theta \;{tan}^{2}\theta }+1\right\} \end{array}$$

Since the parameters $R_{U}$, θ, are either known or measured for the respective wavelength, they can be substituted into Equation (9) to yield equations involving $n_{2}$ and $k_{2}$. By solving these equations simultaneously, the values of $n_{2}$ and $k_{2}$ were determined.

### 4.2. Multi-Layer Absorption Model for Determining $n_{1}$ and $k_{1}$ for the Doped SiC Region

To determine $n_{1}$ and $k_{1}$ for the doped SiC region, where ${\tilde{n}}_{1}$ = $n_{1r}+ik_{1i}$, we apply Snell’s law to the schematic in Figure 3, yielding the following:

(10) $$n_{0}\;\sin\theta_{0}={\tilde{n}}_{1}\;sin{\tilde{\theta }}_{1}={\tilde{n}}_{2}\;sin{\tilde{\theta }}_{2}=n_{0}\;\sin\theta_{3}$$

As per Equation (10), $\theta_{0}=\theta_{3}$.

The perpendicular and parallel components of r for each interface shown in Figure 3 can be expressed as follows [23]:

(11) $${\tilde{r}}_{s}^{\left(ab\right)}=\frac{{\tilde{n}}_{a}\;cos{\tilde{\theta }}_{a}-{\tilde{n}}_{b}\;cos{\tilde{\theta }}_{b}}{{\tilde{n}}_{a}\;cos{\tilde{\theta }}_{a}+{\tilde{n}}_{b}\;cos{\tilde{\theta }}_{b}}$$

(12) $${\tilde{r}}_{p}^{\left(ab\right)}=\frac{{\tilde{n}}_{b}\;cos{\tilde{\theta }}_{a}-{\tilde{n}}_{a}\;cos{\tilde{\theta }}_{b}}{{\tilde{n}}_{b}\;cos{\tilde{\theta }}_{a}+{\tilde{n}}_{a}\;cos{\tilde{\theta }}_{b}}$$

In Equations (11) and (12), the subscript ‘a’ refers to the incident medium, while ‘b’ denotes the transmitted medium. The superscript ‘ab’ represents the interface between two media, such as air–doped region (interface: 01), doped–undoped region (interface: 12), and undoped region–air (interface: 23), as illustrated in Figure 3.

The propagation factor for plane waves is provided by Equation (13) [23]:

(13) $${\tilde{\delta }}_{m}=\frac{2\pi \;{\tilde{n}}_{m}\;d_{m}\;cos{\tilde{\theta }}_{m}}{\lambda_{0}}$$

Here, $\lambda_{0}=4.3\;µm$, with ‘m’ representing either 1 or 2 to indicate media 1 or 2, respectively. ‘d’ denotes the thickness, and ‘θ’ is the incident angle for the corresponding medium. The thickness of medium–1 (doped SiC region), denoted as $d_{1}$ is shown in Figure 3. The value of $d_{1}$ was measured to be 450 nm using Secondary Ion Mass Spectrometry (SIMS) (see Section 5.3).

The perpendicular and parallel components of r across the entire SiC substrate can be expressed as follows [23]:

(14) $${\tilde{r}}_{s}=\frac{\left({\tilde{r}}_{s}^{\left(01\right)}+{\tilde{r}}_{s}^{\left(12\right)}e^{i2{\tilde{\delta }}_{1}}\right)+\left({\tilde{r}}_{s}^{\left(01\right)}{\tilde{r}}_{s}^{\left(12\right)}+e^{i2{\tilde{\delta }}_{1}}\right){\tilde{r}}_{s}^{\left(23\right)}e^{i2{\tilde{\delta }}_{2}}}{\left(1+{\tilde{r}}_{s}^{\left(01\right)}{\tilde{r}}_{s}^{\left(12\right)}e^{i2{\tilde{\delta }}_{1}}\right)+\left({\tilde{r}}_{s}^{\left(12\right)}+{\tilde{r}}_{s}^{\left(01\right)}e^{i2{\tilde{\delta }}_{1}}\right){\tilde{r}}_{s}^{\left(23\right)}e^{i2{\tilde{\delta }}_{2}}}$$

(15) $${\tilde{r}}_{p}=\frac{\left({\tilde{r}}_{p}^{\left(01\right)}+{\tilde{r}}_{p}^{\left(12\right)}e^{i2{\tilde{\delta }}_{1}}\right)+\left({\tilde{r}}_{p}^{\left(01\right)}{\tilde{r}}_{p}^{\left(12\right)}+e^{i2{\tilde{\delta }}_{1}}\right){\tilde{r}}_{p}^{\left(23\right)}e^{i2{\tilde{\delta }}_{2}}}{\left(1+{\tilde{r}}_{p}^{\left(01\right)}{\tilde{r}}_{p}^{\left(12\right)}e^{i2{\tilde{\delta }}_{1}}\right)+\left({\tilde{r}}_{p}^{\left(12\right)}+{\tilde{r}}_{p}^{\left(01\right)}e^{i2{\tilde{\delta }}_{1}}\right){\tilde{r}}_{p}^{\left(23\right)}e^{i2{\tilde{\delta }}_{2}}}$$

Starting from Equations (11) and (12), the perpendicular and parallel components of reflectance, denoted as $R_{s}$ and $R_{p}$, respectively, can be determined as follows [23]:

(16) $$R_{s}={\tilde{r}}_{s}{\tilde{r}}_{s}^{*}$$

(17) $$R_{p}={\tilde{r}}_{p}{\tilde{r}}_{p}^{*}$$

where ${\tilde{r}}_{s}^{*}$ and ${\tilde{r}}_{p}^{*}$ are complex conjugates of ${\tilde{r}}_{s}$ and ${\tilde{r}}_{p}$, respectively.

The reflectance of the doped SiC region $\left(R_{D}\right)$ is the average of both R_s_ and R_p_, and can be expressed as follows:

(18) $$R_{D}=\frac{R_{s}+R_{p}}{2}$$

Given that the parameters $R_{D}$, θ, $d_{1},\;d_{2}\;and\;\lambda_{0}$ are either known or measured, they can be substituted into Equation (18) to yield equations involving $n_{1}$ and $k_{1}$. By solving these equations simultaneously, the values of $n_{1}$ and $k_{1}$ were determined. Detailed derivations for $n_{1}$ and $k_{1}$ are provided in the Supplementary Materials.

## 5. Results and Discussion

### 5.1. Raman Spectra Analysis

**Figure 4:**
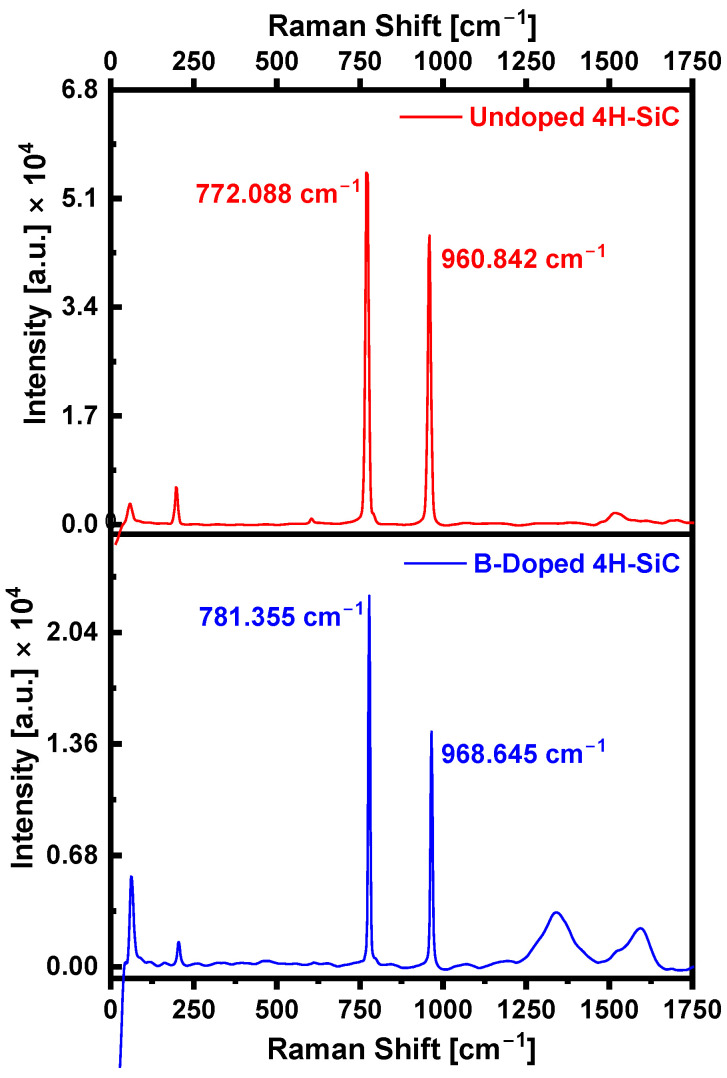
Raman spectra of as-received (red) and boron-doped (blue) 4H-SiC substrates, highlighting the effects of boron incorporation on vibrational modes.

Figure 4 presents the Raman spectra of as-received and boron-doped 4H-SiC substrates obtained using LabRAM HR Evolution Nano (Horiba Instruments Inc., Irvine, CA, USA), revealing distinct modifications in vibrational characteristics resulting from boron incorporation. In the as-received 4H-SiC, the primary Raman-active modes appear at ~772 cm^−1^ (E_2g_) and ~960 cm^−1^ (A_1g_), corresponding to in-plane and out-of-plane vibrations of Si–C bonds, respectively, consistent with the 4H polytype’s crystalline symmetry [26]. Following boron doping, these modes exhibited notable blue shifts (shift to higher wavenumbers) to 781.355 cm^−1^ and 968.645 cm^−1^, respectively. These spectral shifts are indicative of the compressive lattice strain introduced by boron atoms being substituted into the SiC crystal lattice, which alters local bonding configurations by reducing interatomic spacing and increasing bond stiffness, thereby enhancing phonon frequencies [27]. In addition to peak shifts, a reduction in Raman intensity is observed in the doped sample. This attenuation is attributed to the enhanced phonon scattering caused by defect generation and plasmon–phonon coupling, where interactions between free carriers and phonons suppress the Raman scattering efficiency [27]. Moreover, the appearance of broad, additional features in the 1250–1700 cm^−1^ range suggests the presence of disorder-induced vibrational modes, potentially linked to carbon-related phases. Specifically, the observed bands near ~1350 cm^−1^ and ~1580 cm^−1^ correspond to the D-band and G-band of sp^2^-bonded carbon, respectively [28]. These features may arise from local structural disorder, non-stoichiometric carbon clustering, or the formation of graphitic-like domains during the high-temperature laser doping process. These spectral variations highlight the substantial impact of boron doping on the lattice dynamics and structural integrity of 4H-SiC. The observed strain effects, defect signatures, and potential carbon phase formation offer valuable insight into dopant incorporation mechanisms and their influence on the material’s electronic and optoelectronic properties.

### 5.2. Temperature-Dependent Resistivity and Ionization Energy of Boron in 4H-SiC

**Figure 5:**
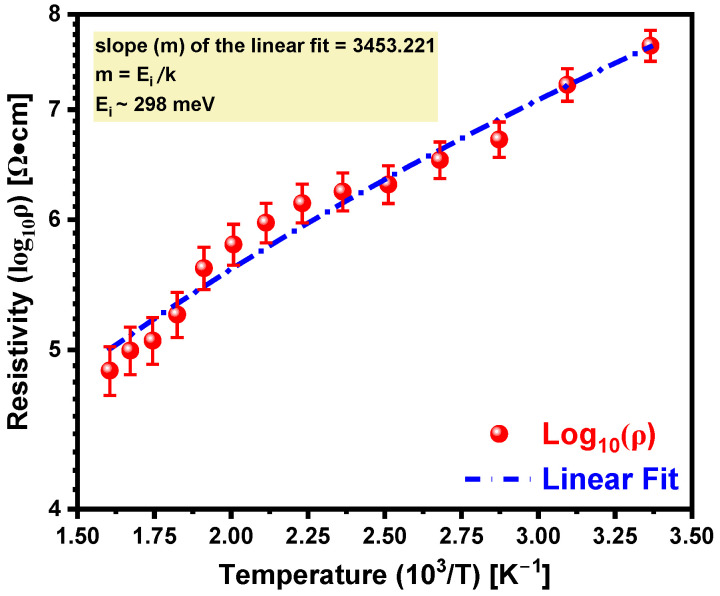
Arrhenius plot of resistivity for boron-doped 4H-SiC. Red circles with standard deviations represent experimentally measured resistivity values across the 300–600 K temperature range, while the dashed blue line shows the linear fit used to determine the slope. The linear behavior of log_10_(ρ) vs. 10^3^/T confirms the thermally activated conduction and enables the extraction of the ionization energy of the boron dopant.

The resistivity (ρ) of boron-doped 4H-SiC can be expressed as $\rho =\rho_{0}\;•\;e^{\left(\frac{E_{i}}{kT}\right)}$ [29], where $\rho_{0}$ is the pre-exponential factor, T is the absolute temperature, k is the Boltzmann constant, and $E_{i}$ is the ionization energy of the boron dopant. Figure 5 illustrates the temperature-dependent resistivity of boron-doped 4H-SiC obtained using P2010A2 Four Point Probe (Ossila Ltd., Sheffield, UK). The slope (m) of the linear fit was determined to be ~3453, which corresponds to $E_{i}$ ~298 meV, as per the relation $E_{i}$ = m • k. The ionization energy in the current study is in good agreement with the previously reported ionization energies for boron acceptors in equilibrium-doped 4H-SiC, which typically range from ~280 to 390 meV [30,31]. However, the extracted value here is particularly significant because doping was achieved via pulsed laser, a non-equilibrium technique. Such laser-induced processes can locally modify the lattice structure through rapid thermal gradients, generate residual point defects, and possibly promote impurity band formation at high dopant concentrations. These effects are known to alter the local electronic structure and reduce the effective ionization barrier for hole-generation from acceptor states. The extracted ionization energy, compared to undisturbed bulk values, suggests the enhanced electrical activation of boron. This implies a higher ionization fraction (f) at room temperature, thereby increasing the availability of free holes for conduction. Such behavior is especially advantageous for p-type conductivity in SiC and can significantly improve the performance of boron-doped layers in p–n junctions, photodetectors, and other SiC-based optoelectronic or power devices.

### 5.3. Secondary Ion Mass Spectrometry (SIMS) Analysis

**Figure 6:**
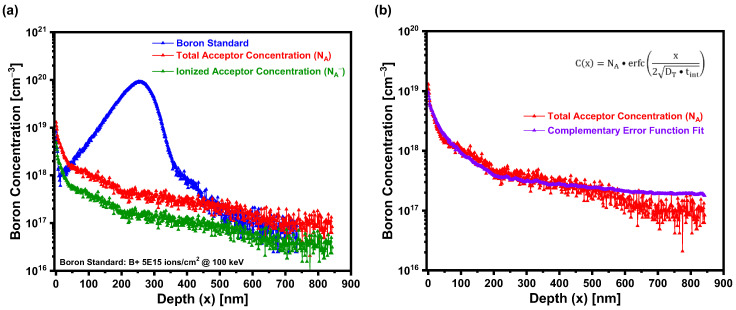
(**a**) SIMS profiles of the laser-doped 4H-SiC substrate, displaying the total and effective acceptor concentrations alongside the boron standard as a function of depth (x). (**b**) The complementary error function (erfc) fit for the total acceptor concentration, yielding a diffusion coefficient of D_T_ = 9.83 × 10^−8^ cm^2^/s.

Figure 6a presents the SIMS depth profile of boron in laser-doped 4H-SiC obtained using PHI Adept 1010 Dynamic SIMS System, showing both the total acceptor concentration $\left(N_{A}\right)$ and the ionized acceptor concentration $\left(N_{A}^{-}\right)$ as functions of depth. The original SIMS data, recorded in arbitrary units, were quantitatively calibrated using a boron-implanted reference sample fabricated through implanting boron ions into n-type 4H-SiC at a dose of $5\times 10^{15}\;ions/{cm}^{2}$ at 100 keV, enabling the conversion of raw SIMS counts to absolute concentrations in units of cm^−3^. A peak boron concentration of 1.29 × 10^19^ cm^−3^ was observed at the surface, followed by a sharp decline to 4.72 × 10^17^ cm^−3^ within the first ~200 nm. This rapid drop was attributed to the substitutional diffusion driven by a steep laser-induced thermal gradient. From 200 to 450 nm, the concentration stabilizes, forming a quasi-plateau, before gradually decreasing, consistent with the knock-on diffusion effects influenced by depth-dependent cooling. $N_{A}^{-}$ as a function of depth was determined using the relation, $N_{A}^{-}=\left(N_{A}\;•\;e^{\frac{-E_{i}}{kT}}\right)$ [29,32]. Figure 6a shows that $N_{A}^{-}$ is slightly lower than $N_{A}$ across the depth profile. This disparity highlights the limited electrical activation of boron in 4H-SiC, consistent with prior findings [1,15,18].

Figure 6b presents the measured total acceptor concentration $\left(N_{A}\right)$ profile in laser-doped 4H-SiC (red curve), obtained via SIMS, alongside a theoretical fit (purple curve) using the complementary error function (erfc) solution to the one-dimensional diffusion equation. The fitting curve employs the analytical solution, $C\left(x\right)=N_{A}\;•\;erfc\left(\frac{x}{2\sqrt{D_{T}\;•\;t_{int}}}\right)$ [33], where D_T_ is the diffusion coefficient and t_int_ = 1 s. The fit analysis yielded a diffusion coefficient of D_T_ = 9.83 × 10^−8^ cm^2^/s, which is several orders of magnitude higher than the values previously reported for boron diffusion in SiC under equilibrium conditions [30,34]. Roccaforte et al. [30] reported boron diffusivities in the range of 5.5 × 10^−15^ cm^2^/s at 1800 °C to 10^−11^ cm^2^/s at 2500 °C, while Kubiak et al. [34] documented a lower diffusivity of ~5 × 10^−9^ cm^2^/s for boron in 4H-SiC due to its strong covalent bonding and limited diffusion pathways. The markedly enhanced diffusivity observed in this work was attributed to the non-equilibrium nature of pulsed laser doping, which likely generated transient high-temperature conditions, induced point defects, and possibly led to localized melting or amorphization. These effects facilitated rapid boron incorporation and transport, enabling the efficient formation of shallow, highly doped p-type regions, thereby demonstrating the potential of laser doping for advanced SiC device applications.

**Figure 7:**
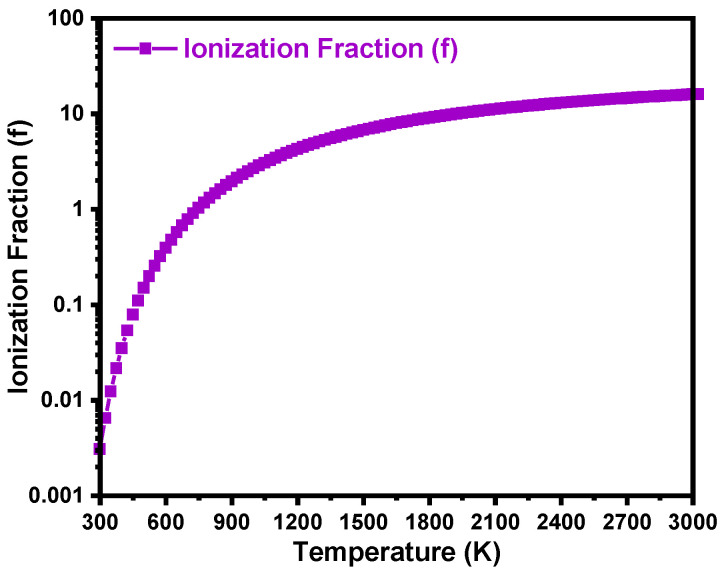
Calculated ionization fraction (f) of boron in 4H-SiC as a function of temperature, based on an ionization energy of 298 meV and a degeneracy factor of 4.

Figure 7 shows the temperature-dependent ionization fraction (f) of boron acceptors in 4H-SiC as a function of temperature (300–3000 K), calculated using $E_{i}$ ~298 meV and acceptor degeneracy factor, $g_{p}$ = 4 [31]. The relationship is given by $f={\left\{1+\left(g_{p}{\;•\;e}^{\frac{E_{i}}{kT}}\right)\right\}}^{-1}$ [29]. The ionization fraction increases steadily with temperature, rising from nearly 0 at 300 K to ~0.164 at 3000 K. This trend reflects the deep acceptor nature of boron in 4H-SiC, where a significant amount of thermal energy is required to activate dopants. Even under elevated thermal conditions (e.g., 2000 K), a comparatively small fraction of boron atoms are ionized. These results highlight the importance of accounting for incomplete ionization when estimating the electrically active dopant concentrations $\left(N_{A}^{-}={f\;•\;N}_{A}\right)$ and when modeling temperature-dependent electrical or optical behavior. The observed ionization characteristics are consistent with prior reports on boron activation in wide-bandgap semiconductors [1,15,18,31].

### 5.4. Current–Voltage Characteristics of the Laser-Fabricated p–n Junction

**Figure 8:**
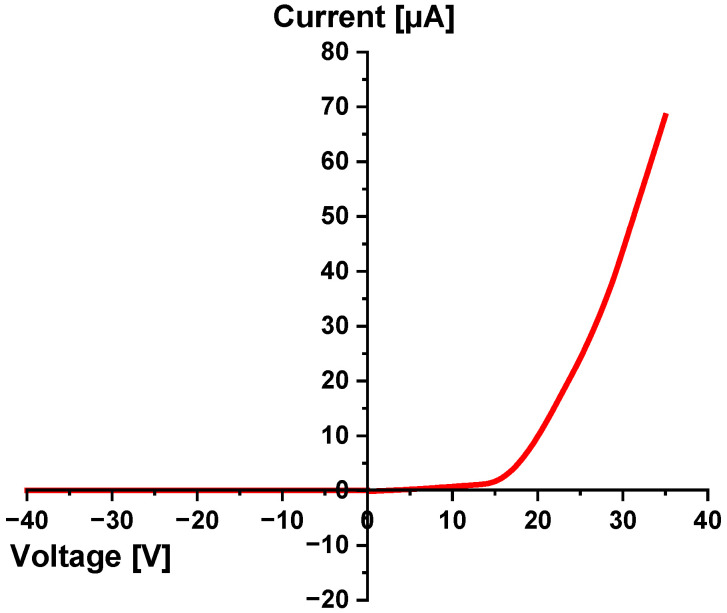
I-V characteristics of a laser-fabricated boron-doped p–n junction in n-type 4H-SiC.

The electrical behavior of the laser fabricated p–n junction diode on 4H-SiC substrate at ambient temperature was assessed using current–voltage (I–V) measurements under both forward- and reverse-bias conditions, performed with a 2450 SourceMeter (Keithley Instruments, Inc., Solon, OH, USA). The I–V plot in Figure 8 demonstrates the classical rectifying behavior of a typical diode, with a negligible leakage current under reverse bias and a rapid exponential increase in current under forward bias. This behavior is consistent with the formation of a well-defined p–n junction and confirms the diode’s unidirectional conduction property [35,36]. Under forward bias, the current begins to increase significantly beyond ~15 V, marking the turn-on voltage. This relatively high turn-on threshold is attributed to the wide bandgap of 4H-SiC (~3.23 eV) [15], which requires a higher voltage to inject sufficient carriers across the junction. As the forward voltage increases beyond 15–20 V, the current rises steeply, reaching ~69 µA at 35 V.

In the reverse-bias region, the diode demonstrates excellent blocking capabilities, with a reverse-leakage current of ~0.2 nA across the voltage range up to −40 V. There is no sharp increase in current in the negative voltage region up to −40 V. Hence, no breakdown is observed within this range, suggesting that the diode has a reverse-breakdown voltage well beyond −40 V. The theoretical breakdown voltage ${(V}_{BR})$ for the laser-fabricated p–n junction in 4H-SiC was calculated using the expression, $V_{BR}=\frac{\varepsilon_{s}E_{C}^{2}}{2qN_{D}}$, where $\varepsilon_{s}$ (dielectric permittivity of 4H-SiC) = 9.7 $\varepsilon_{0}$, $E_{C}$ (critical electric field for 4H-SiC) = 2.5 × 10^6^ V/cm for moderate doping, q is the electronic charge and $N_{D}$ = 10^16^ cm^−3^. Substituting these values, the resulting $V_{BR}$ was found to be 1668 V. This value represents the ideal avalanche breakdown condition for a one-sided abrupt (p^+^–n) junction. This aligns with the expectations for SiC-based diodes, which are known for their high breakdown fields and minimal reverse leakage [35,36]. The built-in potential $\left(V_{bi}\right)$ was estimated using the standard expression, $V_{bi}=\frac{kT}{q}ln\left(\frac{{N_{A}•N}_{D}}{n_{i}^{2}}\right)$, where N_A_ and N_D_ represent the acceptor and donor concentrations, $n_{i}$ is the intrinsic carrier concentration of 4H-SiC, k is the Boltzmann constant, and T is the room temperature in Kelvin. The N_A_ (average) was determined to be 5.41 × 10^17^ cm^−3^ from the SIMS measurements (see Section 5.3), whereas $n_{i}$ has a typical value of 8.2 × 10^−9^ cm^−3^ at 300 K [37]. Hence, $V_{bi}$ was calculated to be 2.952 V which aligns with the corresponding values reported in [38,39]. This relatively high built-in potential is characteristic of the wide-bandgap semiconductors and supports efficient charge separation across the junction. The extracted electrical parameters confirm the viability of the laser doping technique for forming functional p–n junctions in SiC substrates. The high turn-on voltage, excellent reverse-blocking behavior, and exponential forward-conduction reinforce the potential of this device for high-voltage and high-temperature power electronics applications.

### 5.5. Determination of Refraction and Attenuation Indices Pre- and Post-Laser Doping

**Figure 9:**
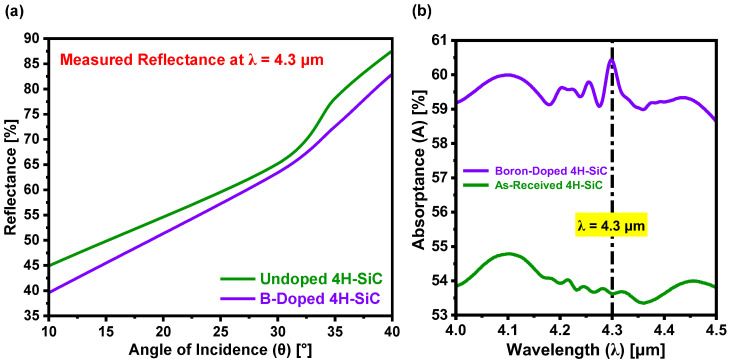
(**a**) Measured reflectance (R) of as-received and boron-doped 4H-SiC substrates at λ = 4.3 µm for angle of incidence (θ), illustrating the influence of boron incorporation on angular reflectance behavior. (**b**) Absorptance (A) of SiC before and after doping, exhibiting a pronounced absorption peak at λ = 4.3 µm attributed to electronic transitions involving the boron-induced acceptor level.

As illustrated in Figure 9a, boron was selected as the p-type dopant for the 4H-SiC substrate due to its ability to introduce a discrete acceptor energy level, E_A_ = 0.29 eV, above the valence band, corresponding to a photon wavelength of λ = 4.3 µm within MIDIR spectrum [40,41]. Upon exposure to electromagnetic radiation at this wavelength, valence band electrons are excited into the boron-induced acceptor level, thereby increasing the electron density ($N_{e}$) and modulating both the refraction index (n) and attenuation index (k) of the doped SiC [1]. This modulation is inherently dependent on the wavelength of the incident electromagnetic radiation and the natural frequency of electrons within the semiconductor [23,42,43], highlighting the potential application of boron-doped 4H-SiC as an MIDIR-responsive material. The optical response before and after doping was evaluated using a PerkinElmer Spectrum-2 FTIR spectrometer (Figure 9a,b). Given that the as-received substrate exhibits negligible transmittance (T~0%) within the MIDIR range, the increase in $N_{e}$ primarily alters the reflectance (R), through which absorptance (A) can be determined as follows: A = 100 − R.

Figure 9a shows the reflectance as a function of incidence angle, revealing a monotonic increase for both samples. Across all measured angles, the boron-doped SiC region $\left(R_{D}\right)$ consistently exhibits lower reflectance compared to the as-received (undoped) SiC region $\left(R_{U}\right)$ of the substrate. This reduction in reflectance is indicative of altered optical constants, particularly the refraction and attenuation indices, due to the introduction of free carriers and impurity states resulting from boron incorporation. The modified optical behavior arises from the enhanced interaction between incident radiation and electronic transitions within the doped semiconductor. The angular reflectance profile further confirms that boron incorporation effectively tailors the surface optical response of 4H-SiC, which is critical for optoelectronic applications in the MIDIR range. Figure 9b presents the corresponding absorptance spectra, where the boron-doped sample exhibits a pronounced absorption peak at λ = 4.3 µm—consistent with transitions from the valence band to the boron-induced acceptor level (E_A_ ~0.29 eV). At λ = 4.3 µm, the absorptance increases from 53.62% in the as-received substrate to 60.43% in the doped sample, confirming successful dopant incorporation. Additionally, a secondary absorption feature emerges near λ ~4.8 µm (corresponding to ~0.258 eV), which may be attributed to interstitial boron, variation in substitutional doping at Si or C lattice sites, or local lattice strain induced by atomic size mismatch [1]. These mechanisms can introduce additional energy levels within the bandgap, enhancing MIDIR absorption. Moreover, the presence of residual impurities or defects within the substrate, possibly activated during the laser doping process, may contribute to this response [1]. To suppress such extrinsic effects, utilizing a high-purity SiC epitaxial layer as the starting substrate is recommended, as epitaxial layers generally contain fewer impurities and defects compared to bulk-grown substrates [1].

**Figure 10:**
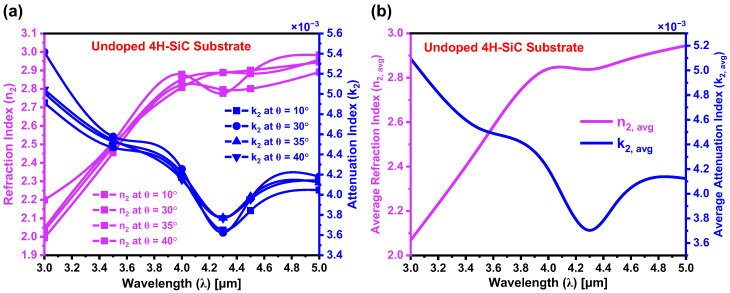
(**a**) Refraction index ($n_{2}$) and attenuation index ($k_{2}$) of the as-received (undoped) region of the 4H-SiC substrate, measured as a function of wavelength (λ) at varying angles of incidence. (**b**) Average values of $n_{2}$ and $k_{2}$ values over the MIDIR spectral range.

As outlined in Section 4, theoretical absorption models were developed to calculate the refraction and attenuation indices of 4H-SiC substrates, both before and after laser doping. Figure 10a presents the refraction index ($n_{2}$) and attenuation index ($k_{2}$) for the as-received (undoped) 4H-SiC substrate at various incident angles. Figure 10b shows the corresponding average values of $n_{2}$ and $k_{2}$ across different angles. At λ=4.3 μm, the extracted average optical constants are $n_{2,avg}=2.857$ and $k_{2,avg}=3.70\times 10^{-3}$. In the visible and NIR regions, $n_{2}$ exhibits a decreasing trend with decreasing wavelength due to the normal dispersion, leading to stronger light-bending. Within the MIDIR region, $n_{2}$ increases due to electronic transitions at shorter wavelengths (~3 μm), resonance effects, phonon interactions at longer wavelengths (~5 μm), and lattice absorption. $k_{2}$ quantifies material absorption and is predominantly governed by free carrier absorption (FCA) and strong electronic transitions. Electronic transitions occur when electrons move between energy levels in a material due to photon absorption. Here, strong electronic transitions refer to the highly probable and efficient excitations of electrons within the band structure of 4H-SiC, particularly around ~3 μm. At these wavelengths, photon energies are higher, making it easier to excite electrons from the valence band to higher-energy states, including impurity levels introduced by doping. FCA is a phenomenon in which free charge carriers—electrons in the conduction band and holes in the valence band—absorb incident photons, leading to increased optical absorption without requiring interband electronic transitions across the bandgap. Unlike interband absorption, which involves electron excitation from the valence band to the conduction band, FCA occurs within the same band and is strongly dependent on the density and mobility of the free carriers. At ~3 μm, $k_{2}$ is high due to FCA and the strong electronic transitions. However, as the wavelength increases, the photon energy decreases, reducing the efficiency of these electronic transitions and thereby lowering optical absorption. This complex interplay between electronic transitions and FCA defines the spectral behavior of both $n_{2}$ and $k_{2}$, as observed in Figure 10a,b.

**Figure 11:**
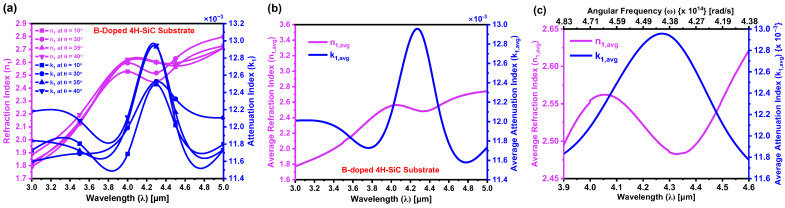
(**a**) Refraction index ($n_{1}$) and attenuation index ($k_{1}$) of the boron-doped region of the 4H-SiC substrate, measured as a function of wavelength (λ) at varying angles of incidence. (**b**) Average values of $n_{1}$ and $k_{1}$ over the MIDIR range plotted against λ; (**c**) Corresponding dependence of the average $n_{1}$ and $k_{1}$ values on the angular frequency (ω), illustrating the dispersion behavior in the frequency domain.

Figure 11a illustrates the variation in the refraction index $\left(n_{1}\right)$ and attenuation index ${(k}_{1})$ for the boron-doped region of the 4H-SiC substrate, extracted using Equation (18) from the absorption models in Section 4. Both $n_{1}$ and $k_{1}$ exhibit notable spectral dependence across the MIDIR range, with measurements taken at multiple angles of incidence. The corresponding average values are presented in Figure 11b, where at λ = 4.3 µm, the extracted values are $n_{1,avg}=2.485$ and $k_{1,avg}=12.94\times 10^{-3}$. The pronounced absorption peak observed in $k_{1}$, particularly near 4.3 µm, reflects enhanced free carrier absorption (FCA) resulting from boron incorporation. The observed decrease in $n_{1}$ across the MIDIR spectrum is primarily attributed to the formation of a boron-induced acceptor level above the valence band of 4H-SiC, which significantly modifies the electronic and optical behavior of 4H-SiC. When boron atoms occupy substitutional sites during laser-induced thermal diffusion, they increase the hole concentration at the acceptor level, thereby elevating the free carrier density. This increase in carrier density intensifies FCA, wherein incident photons are absorbed by charge carriers, and the energy is dissipated through phonon emission. The enhanced FCA leads to increased optical damping, thereby reducing the SiC’s refraction index by diminishing its capacity to sustain coherent electromagnetic wave propagation. Furthermore, the redistribution of charge carriers associated with doping reduces SiC’s electronic polarizability, subsequently decreasing the dielectric constant and contributing to the observed reduction in $n_{1}$. The inset in Figure 11b illustrates the dependence of the average optical constants on angular frequency (ω), further emphasizing the frequency-sensitive nature of carrier-induced absorption and refraction index suppression. These results collectively underscore the complex interplay between boron-induced electronic structure modifications, dielectric behavior, and optical response in 4H-SiC.

**Figure 12:**
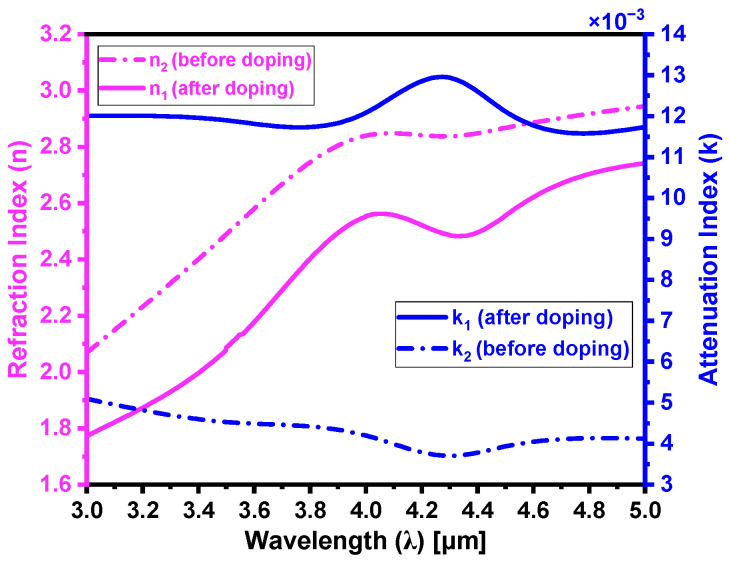
Comparison of n and k for the 4H-SiC substrate before and after laser doping. The dashed curves represent the undoped (as-received) region, while the solid curves correspond to the boron-doped region of the substrate.

Figure 12 provides a comprehensive comparison of the n and k values, emphasizing the changes that occur before and after laser doping. This clearly demonstrates how the optical properties are modified as a result of the incorporation of the boron atoms into SiC, offering a visual depiction of the variations in both the real (n) and imaginary (k) components of the complex refraction index. Table 1 provides a quantitative comparison of the refraction index deviations (Δn) and attenuation index deviations (Δk) between the as-received (undoped) and boron-doped regions. These deviations were calculated using both Fresnel’s method [21] and the absorption models detailed in Section 4. The Δn and Δk obtained using both methods are minimal, with error margins of ~0.016 and $\sim 0.46\times 10^{-3}$, respectively, thereby validating the accuracy of our absorption models.

### 5.6. Determination of Dielectric Constant to Study Anomalous Dispersion in SiC

**Figure 13:**
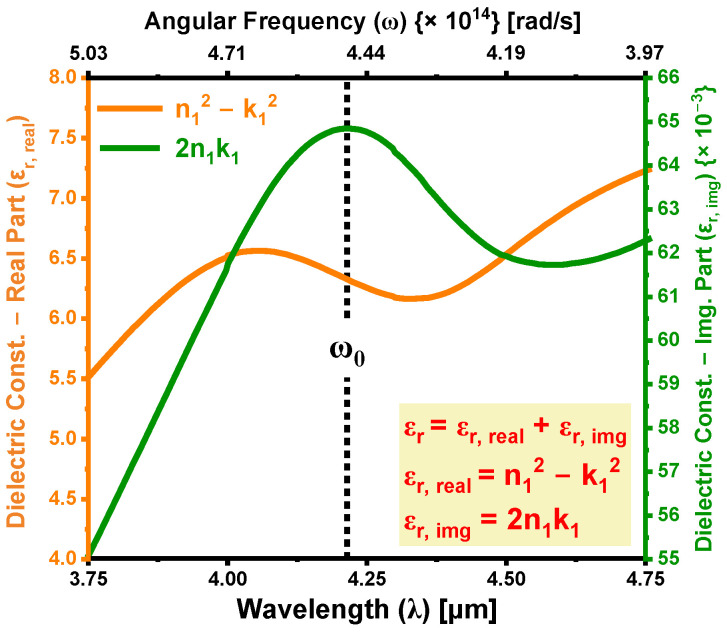
Dispersion curves showing the variation in the real and imaginary parts of the dielectric constant ($\varepsilon_{r}$) as functions of wavelength (λ) and angular frequency (ω).

The variations in the complex permittivity $\varepsilon_{r}$, defined as $\varepsilon_{r,real}=n_{1}^{2}-k_{1}^{2}$ (real part) and $\varepsilon_{r,img}=2n_{1}k_{1}$ (imaginary part), with respect to angular frequency (ω) are depicted in Figure 13. Under typical conditions, $\varepsilon_{r}$ tends to increase as ω increases, a behavior referred to as normal dispersion [23,42]. This trend occurs because, at higher frequencies, the material’s ability to polarize in response to the electric field enhances, leading to an increased dielectric constant. However, an intriguing phenomenon arises in the vicinity of the resonance frequency $\omega_{0}$, where the relationship between $\varepsilon_{r}$ and ω reverses, resulting in a decrease in $\varepsilon_{r}$ as ω increases. This behavior is classified as anomalous dispersion. Such anomalous dispersion signifies strong interactions between light and the electronic structure of the material, particularly as light approaches the frequency of resonance. These interactions can result in unique optical characteristics that may enhance the performance of photonic devices. As shown in Figure 13, the width of the anomalous dispersion region is Γ=0.486 µm. Given that this region is quite narrow, $\omega_{0}$ can be effectively equated to $\omega_{m}$, which is the frequency at which $\varepsilon_{r}$ attains its maximum or minimum value [42]. The difference $\left(\omega_{0}-\omega_{m}\right)$ is expressed as ± Γ/2, with $\left|\omega_{0}-\omega_{m}\right|$ representing the half-width of the anomalous dispersion region, with Γ being the full width [23,42]. Grasping the nature of this anomalous dispersion is crucial, as it can significantly influence the optical properties and potential applications of SiC in a range of photonic devices. For example, the distinctive behavior observed near the resonance frequency can be harnessed in applications like nonlinear optics, waveguiding, and optical sensing, where precise control over light propagation is essential. The complex interplay between electronic transitions and the refraction index in relation to anomalous dispersion provides valuable insights for the development of advanced SiC-based devices in the field of photonics.

Boron doping profoundly transforms the electronic band structure and optical behavior of 4H-SiC, playing a pivotal role in advancing its functionality for optoelectronic applications. By introducing extrinsic recombination centers, boron incorporation alters photoluminescence characteristics, with distinct responses governed by dopant concentration and spatial distribution. These modifications reflect substantial changes in the electronic landscape of SiC [40]. Laser doping not only enables controlled boron integration but also facilitates defect engineering within the crystal lattice. The localized thermal energy generated during laser irradiation promotes dynamic annealing, effectively reducing defect densities and enhancing crystalline quality. This dual capability significantly improves both optical and electrical performance. Additionally, the angle of laser incidence emerges as a critical parameter, as it directly influences reflection and attenuation coefficients—factors essential for device efficiency. Variations in the angle of incidence can modulate the optical response, underscoring the importance of precise geometric and optical alignment during device fabrication. Boron doping also impacts carrier concentration and mobility, increasing free carrier density and altering electrical conductivity. These electronic changes are intrinsically linked to modifications in the refraction and attenuation indices, particularly in the infrared region, where pronounced shifts in reflectance occur. Furthermore, the presence of boron introduces impurity-related optical transitions, evident through shifts in photoluminescence peak positions and intensities [40]. A comprehensive understanding of these effects—ranging from dopant-induced band structure alterations to angular dependencies in optical response—is essential for optimizing the optoelectronic performance of 4H-SiC.

## 6. Conclusions

This study presented a detailed investigation into a laser-induced liquid-phase boron-doping method for n-type 4H-SiC, utilizing a pulsed Nd:YAG laser (λ = 1064 nm). Through the precise optimization of laser process parameters, the controlled thermal diffusion of boron was achieved without exceeding the peritectic temperature of SiC, thereby maintaining the crystalline integrity of the substrate. FTIR spectrometry confirmed the creation of a boron-induced acceptor energy level at 0.29 eV above the valence band, significantly modulating the optical response of the doped substrate in the MIDIR range. comprehensive optical characterization revealed a substantial reduction in the refraction index from 2.857 (as-received) to 2.485 (doped) and an increase in the attenuation index from 3.70 × 10^−3^ to 12.94 × 10^−3^ at λ = 4.3 µm. These results were validated through advanced angle-resolved absorption models, which showed strong agreement with the conventional Fresnel-based methods, confirming the accuracy of the developed models. Electrical characterization of the laser-fabricated p–n junction diode further demonstrated the efficacy of the doping technique. The diode exhibited a high reverse-breakdown voltage of 1668 V and an exceptionally low reverse-leakage current (~0.2 nA), indicating excellent rectifying behavior and suitability for high-voltage applications. A junction depth of 450 nm was measured, with SIMS confirming a peak boron concentration of 1.29 × 10^19^ cm^−3^. Notably, the diffusion coefficient (D_T_ = 9.83 × 10^−8^ cm^2^/s) achieved in this study exceeds the values reported under equilibrium conditions by several orders of magnitude, underscoring the advantages of the non-equilibrium laser doping process in enhancing dopant diffusivity. Additionally, the study identified anomalous dispersion behavior in boron-doped 4H-SiC, reflecting strong light–matter interactions and further enhancing its potential for photonic and optoelectronic MIDIR applications. The ability to precisely tailor both optical and electrical properties through this laser doping technique positions 4H-SiC as a promising material for next-generation devices in power electronics and infrared sensing.

## Figures and Tables

**Table 1:** Comparison of the refraction index deviations (Δn) and attenuation index deviations (Δk) between the as-received and boron-doped regions of the 4H-SiC substrate.

		Fresnel’s Method			Absorption Models [Current Study]		
Wavelength (λ)	Parameter	As-Received	Boron-Doped	Δ	As-Received	Boron-Doped	Δ
4.3 µm	n	2.873	2.517	0.356	2.857	2.485	0.372
	k	${3.75\times 10}^{-3}$	${12.53\times 10}^{-3}$	${8.78\times 10}^{-3}$	${3.70\times 10}^{-3}$	${12.94\times 10}^{-3}$	${9.24\times 10}^{-3}$

## Data Availability

All data and derivations supporting the findings of this study are provided in the article and its Supplementary Information.

## Supplementary Materials

The following supporting information can be downloaded at: https://www.mdpi.com/article/10.3390/ma18122758/s1.
