# Supplementary material for: Laser-Induced Liquid-Phase Boron Doping of 4H-SiC
Source: Materials (Basel). 2025 Jun 12;18(12):2758. doi: 10.3390/ma18122758 (PMC12194778; doi:10.3390/ma18122758)
Supplement: Supplementary file 1 [file materials-18-02758-s001.zip › materials-3504914-supplementary.pdf]

Laser-Induced Liquid-Phase Boron Doping of 4H-SiC

Gunjan Kulkarni[1,2], Yahya Bougdid[2,3], Chandraika (John) Sugrim[1,4], Ranganathan Kumar[3] and Aravinda Kar[1,2,*]

[1] Electrical and Computer Engineering Department, University of Central Florida, Orlando, FL 32816, USA
[2] Center for Research and Education in Optics and Lasers, University of Central Florida, Orlando, FL 32816, USA
[3] Mechanical and Aerospace Engineering Department, University of Central Florida, Orlando, FL 32816, USA
[4] Naval Air Warfare Center – Aircraft Division (NAWCAD), Patuxent River, MD 20670, USA
*akar@creol.ucf.edu

Supplementary Materials

In Fig. 3 from the manuscript, n_0 denotes the refraction index of air (media, m = 0 and m = 3), n_1 and k_1 denote the refraction and attenuation indices of the doped region (medium, m = 1) of the substrate, and n_2 and k_2 represent the refraction and attenuation indices for the as-received (undoped) region (medium, m = 2) of the substrate. The angles θ_1 and θ_3 are real and known from experimental conditions under oblique incidence, while the angles $\tilde{\theta}_1$ and $\tilde{\theta}_2$ are complex angles.

To determine the refraction index (n_1) and attenuation index (k_1) of the doped region within the SiC substrate where $\tilde{n}_1 = n_{1r} + i\,k_{1i}$, applying Snell's law to the schematic illustration from Fig. 3, we obtain:

$$n_0 \sin\theta_0 = \tilde{n}_1 \sin\tilde{\theta}_1 = \tilde{n}_2 \sin\tilde{\theta}_2 = n_0 \sin\theta_3 \quad (1)$$

Here, n_0 denotes the refraction index of air and as per Eq. 1, $\theta_0 = \theta_3$.

As reported in [1], the perpendicular and parallel components of reflection coefficient for each interface shown in Fig. 3 can be written as follows:

$$\tilde{r}_s^{(ab)} = \frac{\tilde{n}_a \cos\tilde{\theta}_a - \tilde{n}_b \cos\tilde{\theta}_b}{\tilde{n}_a \cos\tilde{\theta}_a + \tilde{n}_b \cos\tilde{\theta}_b} \quad (2)$$

$$\tilde{r}_p^{(ab)} = \frac{\tilde{n}_b \cos\tilde{\theta}_a - \tilde{n}_a \cos\tilde{\theta}_b}{\tilde{n}_b \cos\tilde{\theta}_a + \tilde{n}_a \cos\tilde{\theta}_b} \quad (3)$$

Simplifying $\tilde{r}_p^{(ab)}$, we obtain:

$$\tilde{r}_p^{(ab)} = \frac{\tilde{n}_b \cos\tilde{\theta}_a - \tilde{n}_a \cos\tilde{\theta}_b}{\tilde{n}_b \cos\tilde{\theta}_a + \tilde{n}_a \cos\tilde{\theta}_b} = \frac{\tilde{n}_a\tilde{n}_b}{\tilde{n}_a\tilde{n}_b}\left\{\frac{\tilde{n}_b \cos\tilde{\theta}_a - \tilde{n}_a \cos\tilde{\theta}_b}{\tilde{n}_b \cos\tilde{\theta}_a + \tilde{n}_a \cos\tilde{\theta}_b}\right\} = \frac{\tilde{n}_b^{\,2}\,(\tilde{n}_a\cos\tilde{\theta}_a) - \tilde{n}_a^{\,2}(\tilde{n}_b \cos\tilde{\theta}_b)}{\tilde{n}_b^{\,2}\left(\tilde{n}_a\cos\tilde{\theta}_a\right) + \tilde{n}_a^{\,2}\,(\tilde{n}_b \cos\tilde{\theta}_b)} \quad (4)$$

In Eqs. 2 and 3, the subscript 'a' denotes the incident medium whereas subscript 'b' denotes the transmitted medium. The superscripts 'ab' represent the interface between two media, such as the air–doped region (interface: 01), the doped region–undoped region (interface: 12), and the undoped region–air (interface: 23), as shown in Fig. 3.

For simplification, the terms $\left(\tilde{n}_a \cos\tilde{\theta}_a\right)$ and $(\tilde{n}_b \cos\tilde{\theta}_b)$ from Eqs. 2 and 3 are expressed in their real and imaginary parts as follows:

$$\tilde{n}_a \cos\tilde{\theta}_a = A_a + i\,B_a \quad (5)$$

$$\tilde{n}_b \cos\tilde{\theta}_b = C_b + i\,D_b \quad (6)$$

Determination of real and imaginary parts appearing in Eqs. 5 and 6:

$$\tilde{r}_s^{(ab)} = \frac{\tilde{n}_a \cos\tilde{\theta}_a - \tilde{n}_b \cos\tilde{\theta}_b}{\tilde{n}_a \cos\tilde{\theta}_a + \tilde{n}_b \cos\tilde{\theta}_b} = \frac{A_a + i\,B_a - (C_b + i\,D_b)}{A_a + i\,B_a + (C_b + i\,D_b)} = \frac{(A_a - C_b) + i\,(B_a - D_b)}{(A_a + C_b) + i\,(B_a + D_b)} \quad (7)$$

According to the formula for dividing complex numbers, the above expression can be written as:

$$\tilde{r}_s^{(ab)} = \frac{(A_a - C_b)\,(A_a + C_b) + (B_a - D_b)(B_a + D_b)}{(A_a + C_b)^2 + (B_a + D_b)^2} + i\,\frac{(B_a - D_b)(A_a + C_b) - [(A_a - C_b)(B_a + D_b)]}{(A_a + C_b)^2 + (B_a + D_b)^2}$$

The real and imaginary parts of $\tilde{r}_s^{(ab)}$ can be written as:

$$\tilde{r}_s^{(ab)} = \left(\frac{(A_a^2 - C_b^2) + (B_a^2 - D_b^2)}{(A_a + C_b)^2 + (B_a + D_b)^2}\right) + i\left(\frac{(B_a - D_b)(A_a + C_b) - [(A_a - C_b)(B_a + D_b)]}{(A_a + C_b)^2 + (B_a + D_b)^2}\right) = X_s^{(ab)} + i\,Y_s^{(ab)} \quad (8)$$

Where,

$$X_s^{(ab)} = \left(\frac{(A_a^2 - C_b^2) + (B_a^2 - D_b^2)}{(A_a + C_b)^2 + (B_a + D_b)^2}\right) \quad (9)$$

$$Y_s^{(ab)} = \left(\frac{(B_a - D_b)(A_a + C_b) - [(A_a - C_b)(B_a + D_b)]}{(A_a + C_b)^2 + (B_a + D_b)^2}\right) \quad (10)$$

Similarly, $\tilde{r}_p^{(ab)}$ can be expressed as,

$$\tilde{r}_p^{(ab)} = X_p^{(ab)} + i\,Y_p^{(ab)}$$

The expressions for A_a, B_a, C_b and D_b can be obtained as follows:

$$\tilde{n}_a \cos\tilde{\theta}_a = A_a + i\,B_a$$

$$\tilde{n}_a \cos\tilde{\theta}_a = \tilde{n}_a \sqrt{1 - \sin^2\theta_a} = A_a + i\,B_a$$

Snell's law, $\tilde{n}_a \sin\tilde{\theta}_a = n_0 \sin\theta_0$ is applied to the undoped region-air interface in order to determine $(\tilde{n}_a \sin\tilde{\theta}_a)$.

$$\tilde{n}_a \cos\tilde{\theta}_a = \tilde{n}_a \sqrt{1 - \sin^2\theta_a} = \tilde{n}_a \sqrt{1 - \left(\frac{n_0 \sin\theta_0}{\tilde{n}_a}\right)^2}$$

The refraction index of medium 'a' can be expressed as, $\tilde{n}_a = n_a + i\,k_a$ (11)

$$\tilde{n}_a \cos\tilde{\theta}_a = A_a + i\,B_a = \sqrt{\{n_a^2 - k_a^2 - (n_0 \sin\theta_0)^2\} + i\,\{2\,n_a\,k_a\}}$$

Using the formula for the square root of a complex number, the expression above can be rewritten as follows:

$$\tilde{n}_a \cos\tilde{\theta}_a = A_a + i\,B_a = \pm\frac{1}{\sqrt{2}}\left[\left(\sqrt{\sqrt{\{n_a^2 - k_a^2 - (n_0 \sin\theta_0)^2\}^2 + \{2\,n_a\,k_a\}^2} + \{n_a^2 - k_a^2 - (n_0 \sin\theta_0)^2\}}\right) + i\left(\sqrt{\sqrt{\{n_a^2 - k_a^2 - (n_0 \sin\theta_0)^2\}^2 + \{2\,n_a\,k_a\}^2} - \{n_a^2 - k_a^2 - (n_0 \sin\theta_0)^2\}}\right)\right] \quad (12)$$

Here,

$$A_a = \pm\frac{1}{\sqrt{2}}\left[\left(\sqrt{\sqrt{[n_a^2 - k_a^2 - (n_0 \sin\theta_0)^2]^2 + [2\,n_a\,k_a]^2} + [n_a^2 - k_a^2 - (n_0 \sin\theta_0)^2]}\right)\right] \quad (13)$$

$$B_a = \pm\frac{1}{\sqrt{2}}\left[\left(\sqrt{\sqrt{[n_a^2 - k_a^2 - (n_0 \sin\theta_0)^2]^2 + [2\,n_a\,k_a]^2} - [n_a^2 - k_a^2 - (n_0 \sin\theta_0)^2]}\right)\right] \quad (14)$$

Similarly, for $(\tilde{n}_b \cos\tilde{\theta}_b) = C_b + i\, D_b$, we obtain:

$$C_b = \pm\frac{1}{\sqrt{2}}\left[\left(\sqrt{\sqrt{[n_b^2 - k_b^2 - (n_0 \sin\theta_0)^2]^2 + [2\, n_b\, k_b]^2} + [n_b^2 - k_b^2 - (n_0 \sin\theta_0)^2]}\right)\right] \tag{15}$$

$$D_b = \pm\frac{1}{\sqrt{2}}\left[\left(\sqrt{\sqrt{[n_b^2 - k_b^2 - (n_0 \sin\theta_0)^2]^2 + [2\, n_b\, k_b]^2} - [n_b^2 - k_b^2 - (n_0 \sin\theta_0)^2]}\right)\right] \tag{16}$$

The propagation factor for the plane waves can be expressed as [1]:

$$\tilde{\delta}_m = \frac{2\pi\tilde{n}_m d_m \cos\tilde{\theta}_m}{\lambda_0} \tag{17}$$

Here, $\lambda_0 = 4.3$ µm, with 'm' representing either 1 or 2 to indicate media 1 or 2, respectively (Fig. 3). 'd' is the thickness and 'θ' incidence angle for the corresponding medium. The SiC substrate has a thickness of D = 330 µm. The thickness of medium-1 (doped region of SiC) denoted as 'd_1' is shown in Fig. 3. The value of d_1 was measured to be 450 nm using Secondary Ion Mass Spectrometry (SIMS), as presented in Fig. 6(a) of the manuscript. The reflection coefficient components, both perpendicular and parallel, for the entire SiC substrate (across all media) can be expressed as follows [1]:

$$\tilde{r}_s = \frac{\left(\tilde{r}_s^{(01)} + \tilde{r}_s^{(12)}\, e^{i2\tilde{\delta}_1}\right) + \left(\tilde{r}_s^{(01)}\, \tilde{r}_s^{(12)} + e^{i2\tilde{\delta}_1}\right) \tilde{r}_s^{(23)}\, e^{i2\tilde{\delta}_2}}{\left(1 + \tilde{r}_s^{(01)}\, \tilde{r}_s^{(12)} e^{i2\tilde{\delta}_1}\right) + \left(\tilde{r}_s^{(12)} + \tilde{r}_s^{(01)}\, e^{i2\tilde{\delta}_1}\right) \tilde{r}_s^{(23)}\, e^{i2\tilde{\delta}_2}} \tag{18}$$

$$\tilde{r}_p = \frac{\left(\tilde{r}_p^{(01)} + \tilde{r}_p^{(12)}\, e^{i2\tilde{\delta}_1}\right) + \left(\tilde{r}_p^{(01)}\, \tilde{r}_p^{(12)} + e^{i2\tilde{\delta}_1}\right) \tilde{r}_p^{(23)}\, e^{i2\tilde{\delta}_2}}{\left(1 + \tilde{r}_p^{(01)}\, \tilde{r}_p^{(12)} e^{i2\tilde{\delta}_1}\right) + \left(\tilde{r}_p^{(12)} + \tilde{r}_p^{(01)}\, e^{i2\tilde{\delta}_1}\right) \tilde{r}_p^{(23)}\, e^{i2\tilde{\delta}_2}} \tag{19}$$

Starting from Eqs. 2 and 3, the perpendicular and parallel components of reflectance, denoted as R_s and R_p, can be calculated as follows:

$$R_s = \tilde{r}_s \tilde{r}_s^* \tag{20}$$

$$R_p = \tilde{r}_p \tilde{r}_p^* \tag{21}$$

Where $\tilde{r}_s^*$ and $\tilde{r}_p^*$ are the respective complex conjugates.

The reflectance of the doped region of the SiC substrate (R_D) can be expressed as the average of both R_s and R_p, as follows:

$$R_D = \frac{R_s + R_p}{2} \tag{22}$$

Given that the parameters R_D, θ, d_1, d_2 and λ_0 are either known or measured, they can be substituted into Eq. 22 to generate Eqs. involving n_1 and k_1. By solving these equations simultaneously, the values of n_1 and k_1 can be determined.

REFERENCE

1. Hecht, Eugene. Optics. Pearson Education India, 2012.
